# Linking Fearfulness and Coping Styles in Fish

**DOI:** 10.1371/journal.pone.0028084

**Published:** 2011-11-30

**Authors:** Catarina I. M. Martins, Patricia I. M. Silva, Luis E. C. Conceição, Benjamin Costas, Erik Höglund, Øyvind Øverli, Johan W. Schrama

**Affiliations:** 1 Centro de Ciências do Mar (CCMAR), Universidade do Algarve, Faro, Portugal; 2 Aquaculture and Fisheries Group, Wageningen University, Wageningen, The Netherlands; 3 Centro Interdisciplinar de Investigação Marinha e Ambiental (CIIMAR), Universidade do Porto, Porto, Portugal; 4 Institute of Aquatic Resources, Section for Aquaculture, North Sea Center, Danish Technical University, Hirtshals, Denmark; 5 Department of Animal and Aquacultural Sciences, Norwegian University of Life Sciences, Ås, Norway; University of Lethbridge, Canada

## Abstract

Consistent individual differences in cognitive appraisal and emotional reactivity, including fearfulness, are important personality traits in humans, non-human mammals, and birds. Comparative studies on teleost fishes support the existence of coping styles and behavioral syndromes also in poikilothermic animals. The functionalist approach to emotions hold that emotions have evolved to ensure appropriate behavioral responses to dangerous or rewarding stimuli. Little information is however available on how evolutionary widespread these putative links between personality and the expression of emotional or affective states such as fear are. Here we disclose that individual variation in coping style predicts fear responses in Nile tilapia *Oreochromis niloticus,* using the principle of avoidance learning. Fish previously screened for coping style were given the possibility to escape a signalled aversive stimulus. Fearful individuals showed a range of typically reactive traits such as slow recovery of feed intake in a novel environment, neophobia, and high post-stress cortisol levels. Hence, emotional reactivity and appraisal would appear to be an essential component of animal personality in species distributed throughout the vertebrate subphylum.

## Introduction

Individual variation in the physiological and behavioural responses to aversive stimuli is increasingly viewed as adaptive responses that are crucial for survival in a continuously changing environment [1]. In contrast to the presumed advantages of flexible responses, when faced with changing environmental conditions, individuals of the same species or population show consistent responses in stressful and dangerous situations [2], [3], [4]. This phenomenon is referred to as animal personality [5], behavioural syndrome [6], temperament [7], or coping style [2]. In general, some individuals show a proactive behavioural pattern, consistently being more aggressive, more explorative, more neophilic, and more actively avoiding danger than their reactive counterparts. In addition to consistent differences in behavioural traits that correlate among each other, proactive and reactive individuals also differ in neuro-endocrine traits. Proactive individuals have a low hypothalamus-pituitary adrenal/ interrenal (HPA, HPI in fish) axis responsiveness, but high sympathetic reactivity, while the opposite is true for reactive individuals [2], [3], [8]. There is evidence that the physiological traits correlated to animal personality are heritable (e.g. [9], [10]), and contrasting personalities are associated with different fitness consequences [5], which suggests that personality is subjected to evolutionary processes. Likewise, emotions are thought to confer survival advantages by giving animals the ability to avoid harm/punishments and seek valuable resources/reward (e.g. [11], [12]). Under an evolutionary point of view, therefore, emotions - by being functional and adaptive - are unlikely to have evolved spontaneously in the recent human lineage. In addition, the capacity for emotions is likely to differ substantially between species as a consequence of both evolutionary lineage and selective pressures associated with life history [13]. Fear, for example, as a negative emotion increases precautionary behaviour, allowing individuals to avoid potential threat or danger and, therefore has an adaptive value [14].

There are indications that certain stimuli are appraised as fearful in a wide variety of animal groups. This has been demonstrated by behavioural responses to direct exposure to novelty and/or predators (e.g. [15]–[19]). Such responses in fish have been used to describe differences in boldness, and have been interpreted in different ways, such as neophobia [19], reduced exploration or hesitancy [17] or emotional reactivity [18] including fearfulness [15], [16]. However, to which extent responses to direct exposure to aversive stimuli involves common phylogenic roots of cognitive processes involved in fear, such as appraisal, is largely unknown.

The link between personality or coping styles and emotions, including fear, has been addressed in humans, non-human mammals and birds. The individual variation in the threshold for when a stimulus becomes inhibiting rather than stimulatory, i.e. coping style (sensu [2]) is likely correlated to the individual's subjective experience of that stimulus in a given situation. Different personality types have been shown to differ in emotional reactivity [20], the reactivity to negative appraisals [21] and susceptibility to psychological illness [22]. Fear reactivity, for example, has been shown to be a dimension of temperament in humans [23], [24] influencing the susceptibility to depression and anxiety [25]. However, how evolutionary widespread these putative links between personality and the expression of fear are remains to be studied.

Utilizing a teleost fish as a comparative vertebrate model allows investigation of the link between emotions and endocrinal and behavioural dimensions of coping styles in this animal group. Further, this will add to our understanding of the evolutionary relevance and adaptive value of personality, and unravel whether emotions are an essential component of coping styles in species distributed throughout the vertebrate subphylum.

We investigated whether coping styles can predict fear responses in fish using the principle of avoidance learning (combination of classical and operant conditioning). Fish previously screened along the proactive-reactive styles continum (using 3 subsequent tests: feed recovery after transfer itno a novel environemnt, novel object and net restraining) were given the possibility to escape an aversive stimulation that was associated with a cue signalling the onset of the aversive stimuli. In this study, individuals of Nile tilapia were subjected to a signaled aversive stimulus for 7 days (conditioned stimulus, CS: stopping water inflow for 30 sec; unconditioned stimulus, US: confinement stress by lowering a frame into the tank until touching the dorsal fin). Afterwards fish were exposed to the CS only and were allowed to escape from the previous confinement area by using an escape door. The individual variation in escape behavior in this fish was registered and related with the behavior and neuro-endocrine profiling of the same fish screened for coping styles.

Nile tilapia, *Oreochromis niloticus* was used as a model species due to its well characterized behaviour, endocrine and physiological profiles in different behavioural paradigms, including conditioning [26], [27].

## Results

### Coping styles in Nile tilapia

**Figure 1:**
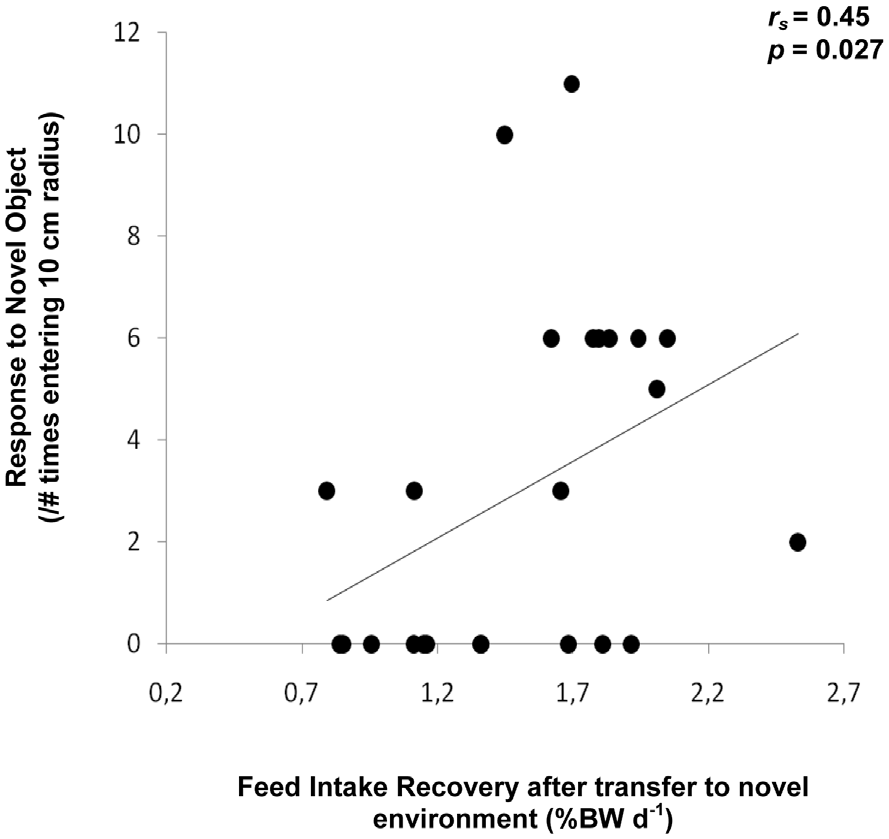
Relationship between feed intake recovery after transfer to a novel environment and neophobia (n = 24).

Feed intake recovery after transfer into a novel environment was shown to predict neophobia (*r_s_* = 0.45, *p* = 0.027, Fig. 1). This suggests that fish recovering their feed intake faster after transfer to a novel environment show lower neophobic response when exposed to a novel object, i.e. traits typically ascribed to bold individuals.

No correlation was however found between cortisol after the net restraining stress, feed intake recovery and the behaviour during the novel object test (*p*>0.05).

### Avoidance learning

**Figure 2:**
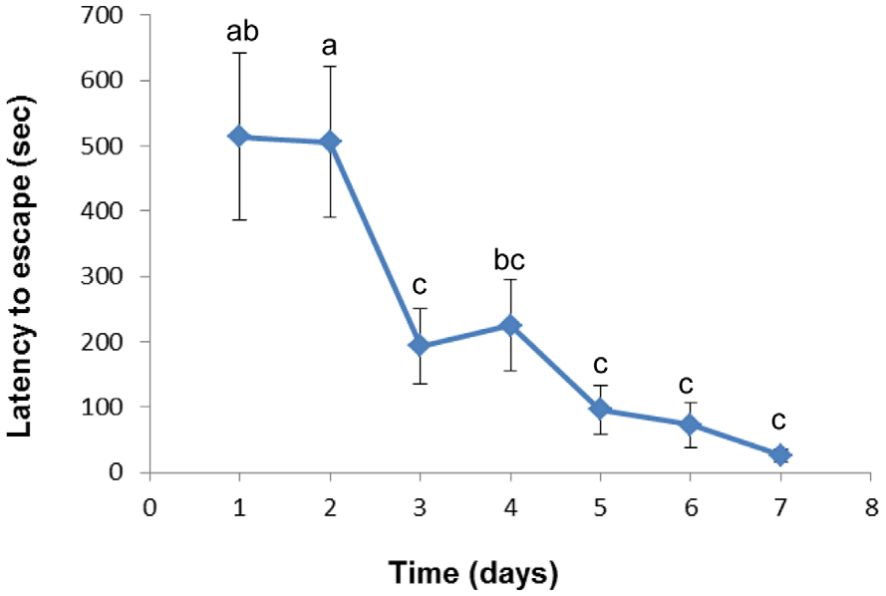
Reduction in latency to escape of *T* fish over the 7 days of CS-US pairing . Each point represents the mean ± SE of 24 individuals. Different letters denote statistical significance at a significant level of *p*<0.05 after repeated ANOVA and Bonferroni comparisons.

Latency to escape from the conditioned stimulus (CS, stopping the water inflow, from now on *water off*) decreased significantly over the 7 days of training (one-way repeated measures ANOVA, *F*_3.10,71.3_ = 14.6, *p*<0.001). On training day 1 fish took, on average, 513 sec to escape, and by day 7 fish were escaping in less than 30 sec (*p* = 0.001, Bonferroni comparison, Fig. 2). During avoidance learning, 22 fish (out of 24) learned to associate the CS (*water off*) with the unconditioned stimulus (US, exposure to a confinement stress); i.e. escaped even in the absence of the confinement frame on day 8. The 2 fish that did not learn were excluded from the analysis concerning the link between coping styles and avoidance learning. It should be noted, however, that these fish did not represent outlier values in regard to previously measured variables.

**Figure 3:**
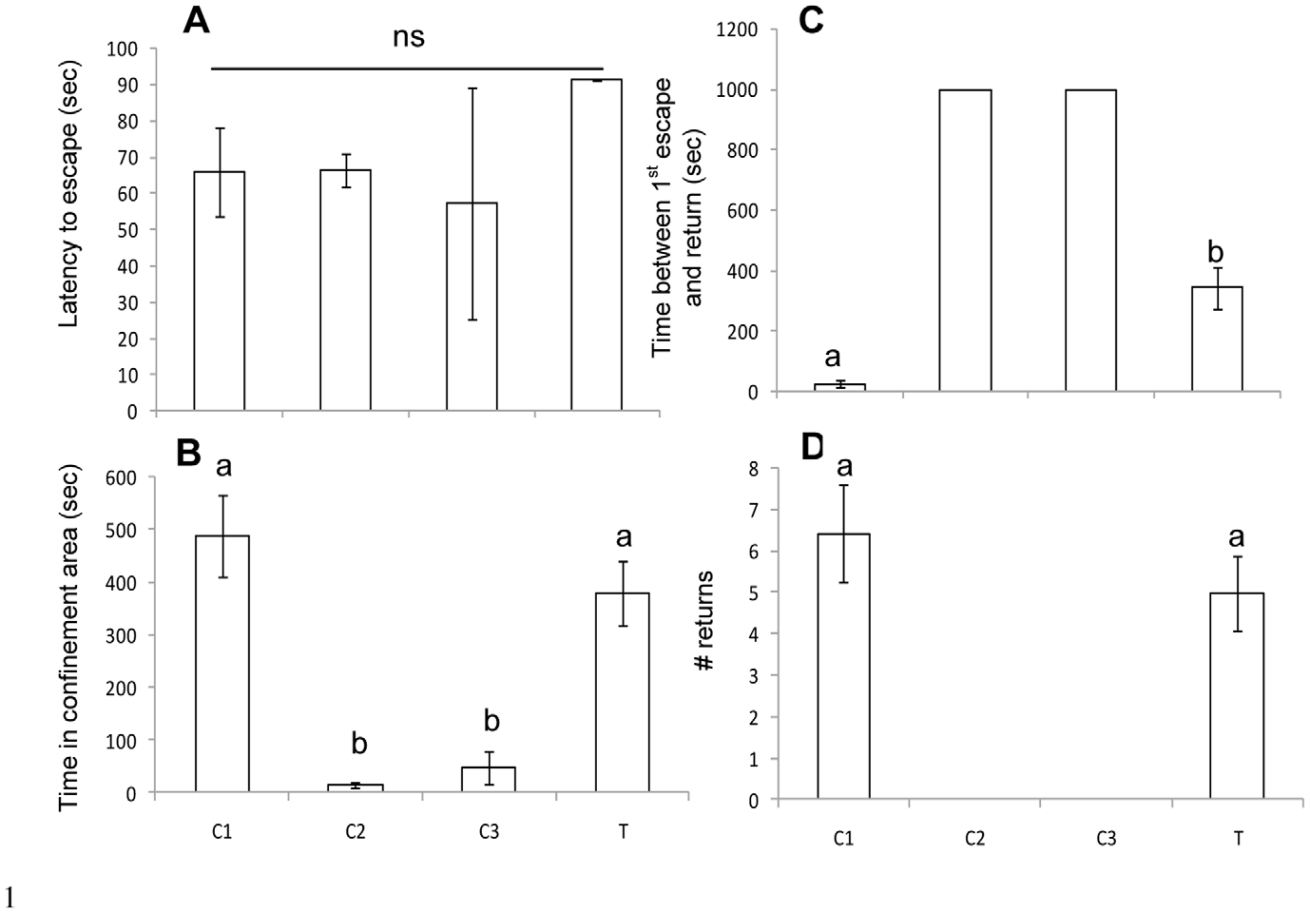
Comparison of escape behavior between *T* and *C1-C3* fish . Latency to escape (A), time spent in confinement area (B), time between 1^st^ escape and 1^st^ return to confinement area (C) and total number of returns to confinement area (D) in *C1*–*C3* (n = 6 in *C1* and *C2* and n =  5 in *C3*) and *T* on day 8, after 7 days of training (n = 22, 2 fish did not escape on day 8 and were not included).

Control and treatment fish did not differ significantly in the latency to escape (Fig. 3, *p*>0.05, Kruskall Wallis test). However, when the time between first escape and return is considered (Figure 3C) significant differences were detected (*p*<0.001). Fish exposed to the confinement stressor only (*C2- confinement*) and in combination with *water off* (*C3-water off/confinement*), escaped through the partition door and did not return to the side where the confinement frame was inserted. Fish exposed to *water off* only during the 7 days of training exhibited the lowest time between escaping and returning (25.2±12.09 sec) while fish exposed to water off only on day 8 after 7 days of pairing between *water off* and confinement showed a significantly higher time between escaping and returning (343.9±71.44 sec, *p* = 0.003, Dunn's comparison). The number of returns and time spent in the confinement area was also higher in *C1-water off* (# returns: 6.4±1.3; time spent in confinement area: 488.4±76.6 sec) as compared with *T-learning* (# returns: 4.9±0.9; time spent in confinement area: 378.2±61.8 sec) but not significantly different (*p*>0.05).

### The relationship between coping styles and avoidance learning

Fish exposed to *T-learning* showed a pronounced individual variation in escape responses. Individuals that took less time to escape were also the individuals that took longer to return to the side of previous confinement (r_s_ = −0.60, p = 0.009) and spent less time in the confinement area on day 8 (r_s_ = 0.44, p = 0.039) while in addition showing the highest cortisol levels in the end of the avoidance learning test (*r_s_* = −0.44, *p* = 0.045), suggesting that fish escaping faster, taking longer to return and spending less time in the confinement area were more stressed even in the absence of the confinement frame.

Time to return after escaping was shown to be correlated positively to cortisol level after the net restraining stress applied on day 35 (*r_s_* = 0.60, *p* = 0.009, Table 1). On the contrary, individuals returning more often to the area of previous confinement (number of returns) and spending more time in that area, exhibited typical characteristics of bold individuals such as lower cortisol response after net restraining (r_s_ = −0.48, p = 0.025,), higher feed intake after transfer to a novel environment (*r* = 0.44, *p* = 0.041), less neophobia when exposed to a novel object (*r* = 0.54, *p* = 0.01 with number of times entering 10 cm radius and *r* = 0.47, *p* = 0.029 with number of times entering 5 cm radius) and more actively trying to escape when restrained (*r_s_* = 0.58, *p* = 0.005).

**Table 1:** Correlation between variables indicating coping styles and fearfulness.

Coping styles\Fearfulness	Latency to escape (sec)	Time between 1^st^ escape and return (sec)	# returns	Time spent in confinement area (sec)
Plasma cortisol after Net Restraining (ng/ml)	ns	*r_s_* = 0.60*p* = 0.009	ns	*r_s_* = −0.48*p* = 0.025
# escape attempts during Net Restraining	ns	ns	*r_s_* = 0.58*p* = 0.005	ns
FI recovery Novel Environment (%BW d^−1^)	ns	ns	*r_s_* = 0.44*p* = 0.04	ns
# times entering 10 cm radius from Novel Object	ns	ns	*r_s_* = 0.54*p* = 0.01	ns

(n = 22 when considering # of returns and time spent in confinement area − 2 out of the 24 fish did not escape on day 8 - and n = 19 when considering the time between escape and return − 2 out of the 24 fish did not escape on day 8 and 3 fish escaped but never returned to the confinement area).

## Discussion

It is now generally accepted that in fish, individual variation in behaviour and physiology when exposed to environmental challenges, reflect the existence of coping styles [3], [28]. This study showed, for the first time, that Nile tilapia *Oreochromis niloticus,* also exhibits divergent coping styles with proactive individuals being characterized by a faster feed intake recovery after transfer into a novel environment and less neophobic when exposed to a novel object, as compared to reactive individuals. Such behavioural responses to challenges have also been described in other fish species [29]–[35].

In classical conditioning, repeated CS–US pairing results in the acquisition of a behavioural conditioned response (CR). In this study, behavioural conditioned response was observed after fish were exposed to the avoidance learning test. The escape behaviour differed significantly between *C1-water off* and the other controls and *T-learning*, as these fish, despite using the escape door returned very quickly to the side where the inflow water was interrupted. In *C1-watter off*, the use of the escape door is probably more related to exploration than to escape behaviour. Fish exposed to the US both alone or in combination with the CS, escaped to the other side of the tank and never returned during the 15 minutes of observation. Fish exposed to *T-learning* (pairing CS–US for 7 days followed by exposure to CS only on day 8) took longer to return to the area where the confinement frame was previously used as compared to fish exposed to *water off* only. Despite fish in *C1-water off* and *T-learning* were exposed to the same stimuli (*water off*), their behaviour differed significantly suggesting that the way the stimuli was interpreted or appraised also differed. This indicates that Nile tilapia can learn how to avoid aversive stimuli by conditioning. A previous study by [26] showed that Nile tilapia can be conditioned to display a stress response in response to conditioned stimuli. In the present study, in addition to classical conditioning, we allowed fish to escape from the aversive stimuli and the results suggest that Nile tilapia is capable of conditioned avoidance learning.

The reason why fish returned to the area of the tank where the confinement frame has been previously used is not clear. It should be noted that the area used for confinement was also the area used for feeding, therefore, one possibility is that the motivation to feed played a role in returning to a potentially dangerous area.

The concept of avoidance learning has been used to investigate fear in different animal species (e.g. in fish [36], [37]). The emergence of consciousness and feelings in fish has been a matter of intense scientific debate (e.g. [38]–[41]). Some authors [39]–[41] argue that this is not possible because their behaviour is simple and reflexive and they lack a neocortex. Yet, a growing body of evidence related to cognitive [42], neuroanatomic [43], [44] and emotional [36], [37], [45] aspects of fish behaviour provides strong support for the ability to feel in fish. In the present study, the observed differences in escape behaviour between fish exposed to *C1-water off* and *T-learning* suggest that these responses are not merely reflexive in nature but are associated with a subjective interpretation of the stimuli. If a reflexive response would be present one would have expected a similar behavioural response between fish exposed to the same stimulus (in our case, *C1-water off* and *T-learning*), which was not the case.

The way individual fish behaved when exposed to *water off* on day 8 (after 7 days of CS–US pairing) was shown to be correlated with traits indicative of coping styles. This suggests that the individual variation in how negative the CS was interpreted (negative appraisal) depends of an individuals' coping style. The link between coping styles and the subjective experience of stimuli and emotional responses has never been investigated in fish, despite studies showing that both (i.e. coping styles and emotions) are possible in fish. This study showed that fish avoiding the area of previous confinement were the fish exhibiting characteristics usually ascribed to reactive or shy individuals, such as lower feed intake recovery after transfer into a novel environment, more neophobic and higher HPI responsiveness after net restraining as compared to proactive or bold individuals. One possible explanation could be a difference in behaviour flexibility between reactive and proactive individuals, in what proactive individuals would be more flexible and therefore prone to modify learned behaviours (in this case the association between *water off* and the onset of confinement resulting in escaping behaviour). This explanation seems, however, unlikely as proactive individuals were shown to be less flexible in modifying learned behaviour than reactive individuals [46]. An alternative explanation is that individuals of the proactive type were less fearful when presented with a signal previously associated with an aversive stimulus, as compared to individuals of the reactive type. Fear is an important component of personality in humans [24], [47], other mammals (e.g., in dogs [48]; in rats [20], [49]) and in birds [50]. The argument for the link between coping styles and fearfulness in fish is evolutionary: fearfulness may be adaptive as it allows individuals to avoid potential threat or danger; from this view, it follows that individual variation in the threshold for when a stimuli becomes inhibitory or stimulatory, i.e. coping style, is likely to be linked with the subjective experience of that stimulus in a particular situation. Severe, chronic and/or unpredictable conditions are likely to provide reactive coping more benefits while mild, intermittent stress and/or predictable conditions are likely to favor proactive responses [51]. Therefore, emotional distress is likely an essential component of reactive coping. This study suggests that the link between coping styles/personality and the expression of emotional or affective states such as fear is an evolutionary widespread phenomenon throughout the vertebrate subphylum, including fish.

This study showed for the first time that cortisol is strongly linked to behaviours indicating fearfulness. A key question that remains to be investigated is whether the link between cortisol responsiveness and fear responses is based on a cause or effect connection. Does the fear reaction potentiate cortisol response, or does elevated cortisol exposure over time alter limbic structures in the brain that mediate fear responses [52]? Further studies are needed to unravel the time course and coordination of psychological and biological stress responses. Extensions of this study could be the investigation of the underlying brain activity in (e.g. through monoamine activity) in differential brain parts, particularly in the medial pallium, an area that is believed to be homologous of the amygdala of land vertebrates [53] and to play an important role in fear responses [54].

This study provides the first evidence that in fish, similarly to what has been found in other vertebrates, individual's coping style is predictive of how stimuli are appraised and the subsequent degree of avoidance behaviour. These results support the inclusion of emotional reactivity and appraisal as essential component of animal personality in species distributed throughout the vertebrate subphylum.

## Materials and Methods

This experiment was approved by the Ethical Committee judging Animal Experiments (DEC no 2009049) of the Wageningen University, The Netherlands.

### Experimental animals, housing and feeding

Forty-two juveniles of Nile tilapia *Oreochromis niloticus* with an initial body weight of 40.8±0.8 g (means±SE) were used as experimental animals. From these, 24 individuals, randomly selected, were used to characterize coping styles and avoidance learning while the remaining fish were used as controls in the avoidance learning test. All animals were obtained from a local tilapia producer (all-male, TilAqua, The Netherlands) where they had experienced common housing and feeding conditions. Upon arrival at Wageningen University, fish were group-housed in a stock tank for 15 days until the start of the experimental procedures. During this period fish were fed *ad libitum* with a commercial diet (2 mm floating pellets; 44% crude protein, 10% fat, 25% carbohydrates, 11.5% ash; Skretting, France) twice a day (08:00 and 16:00) by hand. The same feed was used during the experimental procedures.

During the screening for coping styles (35 days) and avoidance learning (8 days), fish were housed individually in a 40-L glass aquarium (40 cm length×30 cm width×35 cm height, 30 L water capacity, water flow rate was 4 L min^−1^). Tanks were part of a recirculation system operated at a water refreshment rate of 1500 L kg feed^−1^ d^−1^ [55].

Water temperature (26.5±0.1°C), pH (range between 8.6 and 8.7), conductivity (1.96±0.01 mS cm^−1^), TAN (0.05±0.03 mg L^−1^), NO_2_-N (0.00±0.00 mg L^−1^) and NO_3_-N (46.0±2.7 mg L^−1^) were checked daily. A 12 h: 12 h light: dark photoperiod was maintained with daybreak set at 7:00 h.

### Coping styles

Screening for coping styles consisted of subjecting each fish to 3 subsequent tests: 1) novel environment (based on [29], [56]), 2) novel object test (based on [57]) and 3) net restraining test (based on [55]).

The novel environment test consisted of transferring individual fish to a 40-L glass aquarium and following daily feed intake recovery for 14 days. Fish (n = 24) were fed *ad libitum*, by hand, twice per day (08:00 and 16:00) using the same commercial feed as used during the previous 15 days. Feeding continued for a maximum of 1 h, after which the remaining pellets were collected and counted. The average feed intake of the 1^st^ week after transfer to the novel environment was used as indicative of feed intake recovery.

Individually housed fish were kept visually isolated from one another by black plastic around tanks, except for the front side which allowed daily visual observations of the fish.

The novel object test (day 30, after onset of isolation) consisted of a sudden drop of a weighted red LEGO brick (3×3×2 cm, length×width×height) in the middle of the tank, using transparent fishing line attached to the brick to avoid visual contact between the fish and researcher. A mesh screen with squared holes (1 cm) was used on top of the aquarium to allow the determination of the number of times fish entered a 5 and 10 cm radius around the novel object. The latency to enter the 5 cm radius area was also determined using a stopwatch. Fish was considered within the 10 or 5 cm cut-offs when the head was inside that area. The observation period lasted 15 minutes after which the novel object was gently removed.

The net restraining test was conducted on day 35 and consisted of keeping each fish in an emerged net for 60 sec followed by 1 h in the respective tanks (based on [55]). While in the net, the escape behaviour of each fish was determined by counting the number of escape attempts (i.e. body displacements). Blood samples were collected 1 h after the start of net restraining. Fish were rapidly netted and placed in 0.3 g L^−1^ of tricaine methanesulfonate (TMS, Crescent Research Chemicals, Phoenix, Arizona, USA using 0.6 g L^−1^ of sodium bicarbonate as buffer). One mL of blood was collected from all fish by hypodermic syringe (containing 3 mg of Na_2_EDTA) from the caudal blood vessels. This procedure was finalized within 3 min after fish were caught and anaesthetized. The collected blood was placed in cooled 1.5 mL plastic tubes, mixed and centrifuged at 6000×*g* for 5 min at 4°C. After centrifugation plasma was collected and stored at −20°C until cortisol analysis (see below).

### Avoidance learning

**Figure 4:**
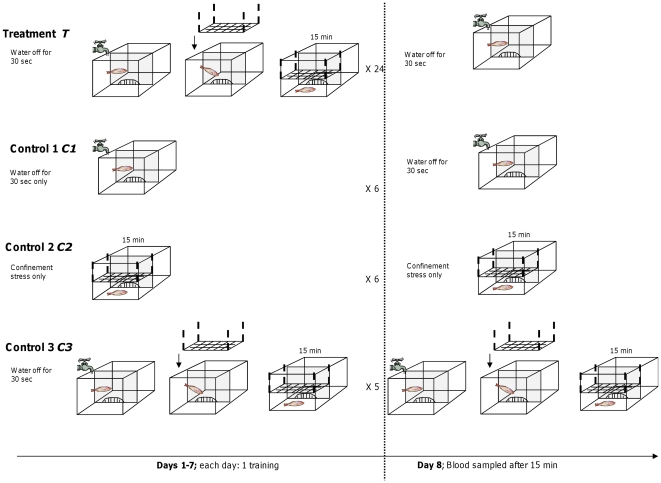
Schematic representation of the experimental set-up used during the avoidance learning test. Fish exposed to avoidance learning (*T-learning*, n = 24) were trained for 7 days to associate *water off* (CS) with the onset of a confinement stress (US) followed by exposure to CS only on day 8. Fish in *C1-water off* (n = 6) were exposed to the CS only, i.e. *water off* during 8 days; Fish in *C2- confinement* (n = 6) were exposed to the US only, i.e., confinement during 8 days without previous signaling by stopping the water inflow; Fish in *C3-water off/confinement* (n = 5) were exposed to CS–US pairing for 8 days. During the 7 days of training the latency to escape was determined. On day 8 in addition to the escape behaviour measures also blood was collected (15 minutes after the start of the US or CS) for cortisol measurements.

After being screened for coping styles each fish was exposed to an avoidance learning paradigm for 8 days (Fig. 4). Four different experimental groups of fish were established: A treatment group (*T- learning,* n = 24) underwent the full avoidance learning test utilising a signalled aversive stimulus (unconditioned stimulus, US). The conditioned stimulus (CS) consisted of stopping the water inflow for 30 sec (from now on *water off*). The US consisted of an iron frame (14 cm×35 cm) lowered into the tank until touching the dorsal fin of the fish, and then remaining there for 15 min. Additionally, 3 different control groups were established (*C1- water off*, *C2-confinement* and *C3- water off/confinement*). Controls were used to test the influence of CS only (*C1*: n = 6 fish were exposed to *water off* once daily during 8 days), US only (*C2*: n = 6 fish were exposed during 8 days to the confinement frame only, without previous signaling) and CS–US pairing (*C3*, n = 5, fish were exposed to CS–US pairing for 8 days, see Figure 1). *C3* and *T* were exposed to the same procedures during 7 days of training, but on day 8, *T* was exposed to CS only while *C3* to CS followed by US.

Each tank was divided in 2 partitions using a PVC divider containing an escape door (half circle, 8 cm diameter) that was opened upon CS presentation. Fish were trained to associate US with CS for 7 days (1 training per day). The latency to escape (i.e. to swim to the side with no confinement frame) was determined daily. In addition to the latency to escape, at this step also the time taken between the first escape and the first return, the total number of returns and the total time spent in the (previous) confinement area, were registered. These behaviours were used as a measure of the degree of responsiveness to a frightening stimulus (based on [36]). After 15 min of observation on day 8 (during this time fish could choose whether and when to return to the previous confinement area), fish were netted and rapidly killed by severing the spinal cord just behind the head. Afterwards, blood (for cortisol analysis) were immediately collected. Blood was processed as described earlier.

Control fish were sampled (for blood), 15 minutes after the start of the US or CS. Fish used in *C1*–*C3* and *T* were all exposed to the experimental conditions prior to the start of the avoidance learning test (however in C1–C3 no coping styles data were collected).

### Analysis of cortisol

Plasma cortisol levels were measured with a commercially available competitive binding Coat-A-Count® Cortisol kit (SIEMENS Medical Solutions Diagnostics, Los Angeles, CA, USA) adapted from [58]. Briefly, 50 µl of each sample to be assayed was transferred into an Ab-Coated tube and 1 ml of ^125^I Cortisol added. The tubes were then incubated for 45 min at 37°C in a water bath. The contents of all tubes were decanted, and allowed to drain for 5 min before being readonagammacounter (2470 WIZARD^2TM^, PerkinElmer^TM^, Inc., Zaventem, Belgium) for 1 min. A calibration curve was constructed on logit-log graph paper and used to convert results from percent binding cortisol to concentration (ng ml^−1^). The Coat-A-Count cortisol antiserum cross-reacts 100% with cortisol, 11.4% with 11-deoxycortisol, 0.98% with cortisone, 0.94% ith corticosterone and 0.02% with progesterone.

### Data analysis

Statistical analyses were performed using SPSS 16.0 for windows. Relationships between variables were investigated using Spearman correlation. To determine whether latency to escape changed over the learning period, a repeated ANOVA (n = 24) was used followed by Bonferroni comparisons. The value of 1000 sec was used when fish did not escape during the 15 minutes observation period. Kruskal Wallis test and Dunn's post-hoc comparison were used to compare the escape behaviour (homogeneity of variances could not be obtained even after data transformation) between controls and treatments. Statistical significance was taken at *p*<0.05.
